# The *PAX3-FOXO1* oncogene alters exosome miRNA content and leads to paracrine effects mediated by exosomal *miR-486*

**DOI:** 10.1038/s41598-019-50592-4

**Published:** 2019-10-02

**Authors:** Farah Ghamloush, Sandra E. Ghayad, Ghina Rammal, Assil Fahs, Abeer J. Ayoub, Zeina Merabi, Mohamad Harajly, Hassan Zalzali, Raya Saab

**Affiliations:** 1 Department of Pediatrics and Adolescent Medicine, Children’s Cancer Institute, American University of Beirut, Beirut, Lebanon; 2 Department of Biology, Faculty of Science II, Lebanese University, Fanar, Lebanon; 3 Department of Anatomy, Cell Biology and Physiology, American University of Beirut, Beirut, Lebanon

**Keywords:** Cell invasion, Paediatric cancer

## Abstract

Rhabdomyosarcoma (RMS) is the most common soft tissue sarcoma in children. The alveolar subtype (ARMS) is clinically more aggressive, and characterized by an oncogenic fusion protein PAX3-FOXO1 that drives oncogenic cellular properties. Exosomes are small, secreted vesicles that affect paracrine signaling. We show that *PAX3-FOXO1* transcript alters exosome content of C2C12 myoblasts, leading to pro-tumorigenic paracrine effects in recipient cells. Microarray analysis revealed alteration in miRNA content of exosomes, affecting cellular networks involved in cell metabolism, growth signaling, and cellular invasion. Overexpression and knockdown studies showed that *miR-486-5p* is an effector of *PAX3-FOXO1*, and mediates its paracrine effects in exosomes, including promoting recipient cell migration, invasion, and colony formation. Analysis of human RMS cells showed *miR-486-5p* is enriched in both cells and exosomes, and to a higher extent in ARMS subtypes. Analysis of human serum samples showed that *miR-486-5p* is enriched in exosomes of patients with RMS, and follow-up after chemotherapy showed decrease to control values. Our findings identify a novel role of both *PAX3-FOXO1* and its downstream effector *miR-486-5p* in exosome-mediated oncogenic paracrine effects of RMS, and suggest its possible use as a biomarker.

## Introduction

Rhabdomyosarcoma (RMS) is the most common soft tissue sarcoma in childhood^1^. The most common histologic subtypes are embryonal (ERMS) and alveolar (ARMS). ARMS tumors account for approximately 20% of RMS^2^, and are characterized by the recurrent reciprocal chromosomal translocation t(2;13), and less frequently t(1;13). The loci involved on chromosomes 1 and 2 encode paired box transcription factors PAX7 and PAX3, respectively; their translocation with chromosome 13 juxtaposes them to the *FOXO1* gene, resulting in a fusion oncoprotein containing the PAX3 or PAX7 DNA binding domain and the C-terminal FOXO1 transactivation domain. Importantly, this oncoprotein has more potent transactivating functions than either PAX3 or PAX7 alone^3^. Clinically, the fusion oncoprotein is an independent negative prognostic marker, and patients with fusion-positive ARMS typically present with advanced disease, and have high rates of tumor recurrence and poorer survival^2,4^.

The role of the fusion oncoprotein PAX3-FOXO1 in RMS cellular behavior has been intensively investigated. PAX3-FOXO1 acts as a transcriptional regulator, affecting a number of genes, in particular those involved in myogenic and developmental processes, proliferation, survival, migration, and metastasis^5–7^. Such downstream effectors of PAX3-FOXO1 include transcription factors such as MYCN^6,8^, growth effectors such as MET^9^, CB1^10^, FGFR4, ALK1, IGF1R, PDGFR-alpha^11,12^, CDKN1B, CDKN1C^13,14^, proteins regulating apoptosis such as Bcl-XL, bcl-2^15,16^, and epigenetic regulators such as JARID2^17^. In addition, *PAX3-FOXO1* was shown to regulate a number of miRNA, to enhance oncologic properties such as invasion and proliferation^18,19^. Importantly, the majority of work has focused on autocrine functions of PAX3-FOXO1 expression, with lack of data regarding effects on paracrine communication.

Paracrine signaling can occur via several mechanisms, including direct secretion of proteins, as well as secretion of microvesicles that can deliver protein, mRNA, and miRNA^20,21^. Exosomes are small vesicles (30–150 nm in size) that are secreted by all cell types, and carry a cargo of proteins, short-chain peptides, lipids, mRNA, and miRNA^22^. By acting on both tumor cells and stroma, exosomes have emerged as new players in tumor invasion, angiogenesis, inflammation and immunologic remodeling^23^. In addition, exosomes have been increasingly studied as possible biomarkers in liquid biopsies of various cancer types^23^.

In this study, we demonstrate that the *PAX3-FOXO1* fusion gene alters the content of exosomes to enhance paracrine signaling that promotes recipient cell invasion, migration, and proliferation. We identified *miR-486-5p* as its downstream effector in exosome-mediated oncogenic paracrine signaling. Examination of human RMS cell lines and patient serum samples confirmed enrichment of *miR-486* in exosomes, suggesting its further investigation as a possible biomarker.

## Results

### *PAX3-FOXO1* expression in C2C12 cells enhances exosome secretion

**Figure 1:**
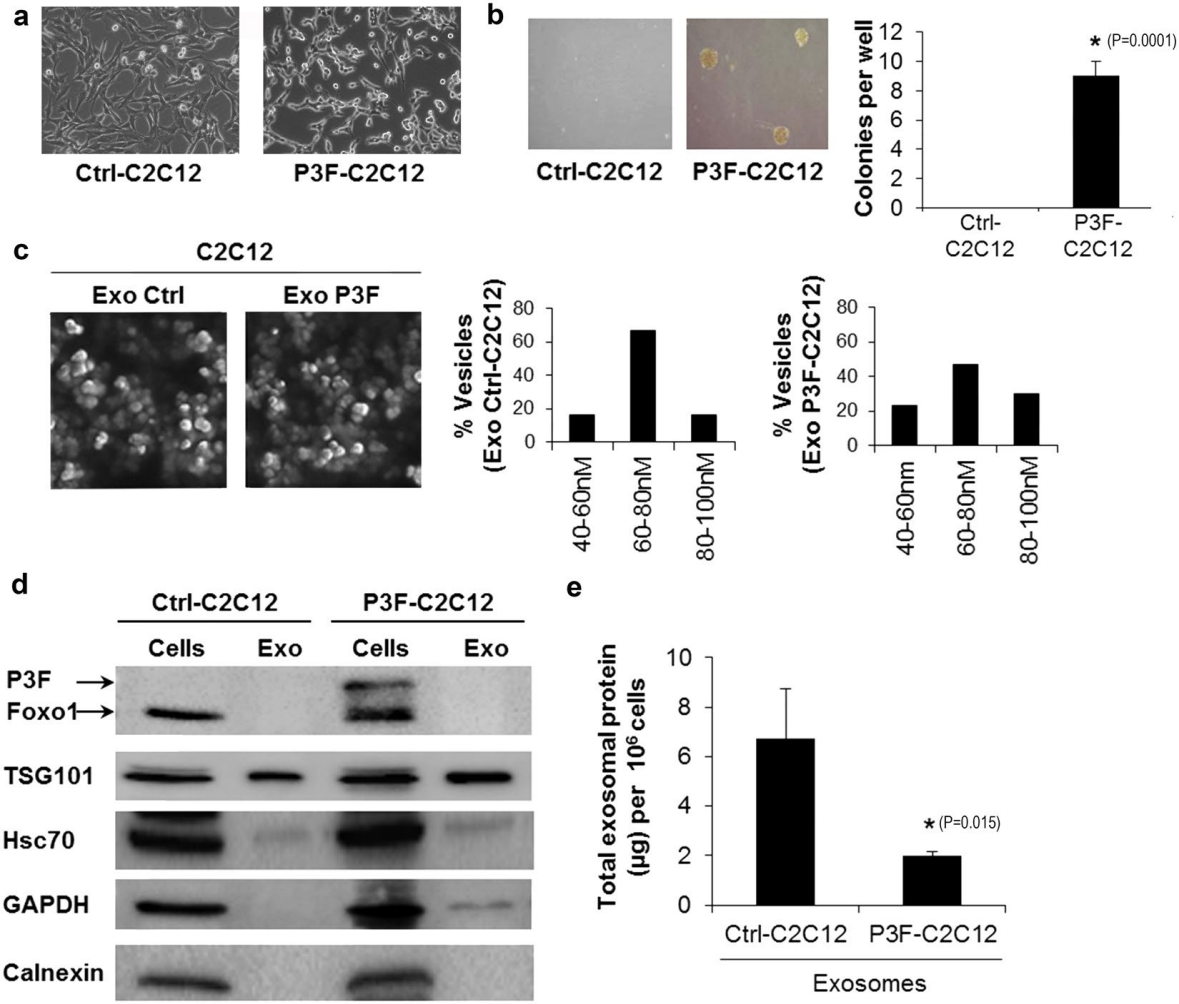
Exosomes are released by P3F-C2C12 and Ctrl-C2C12 mouse myoblasts. (**a**) Representative light microscopy images of C2C12 cells transduced with either empty vector (Ctrl-C2C12) or PAX3-FOXO1 expressing vector (P3F-C2C12). (**b**) Number of colonies formed by the indicated cells in soft agar. Values presented are means of 3 independent experiments and images shown are representative light microscopy pictures of colonies taken after 1 week. (**c**) Representative SEM micrographs of exosomes (Exo) purified from the indicated cells with respective histograms showing the percentage of extracellular vesicles within each diameter range. (**d**) Western Blot analysis of the indicated proteins in exosomes and respective cells. Average exosome proteins (μg) per 1 million cells as quantified by Bradford assay. Bars represent standard deviation. Asterisks denote a statistically significant difference (p-value < 0.05).

We used murine C2C12 myoblasts, a system commonly employed to evaluate cellular effects of *PAX3-FOXO1* in a myogenic precursor background. As expected^10^, *PAX3-FOXO1*-transduced C2C12 cells (P3F-C2C12 cells) showed changes in cellular morphology to a less differentiated phenotype (Fig. 1a), as well as enhanced anchorage-independent growth (Fig. 1b), when compared to empty vector-transduced C2C12 cells (Ctrl-C2C12 cells).

To identify whether *PAX3-FOXO1* expression affected C2C12 exosomes, we extracted exosomes by ultracentrifugation, and verified the nature of extracted vesicles by electron microscopy and size quantification (Fig. 1c), as well as protein analysis showing markers of exosomes such as TSG101, HSC70, and GAPDH, with absence of the endosomal marker Calnexin (Fig. 1d). While the PAX3-FOXO1 protein could be easily identified in the cellular lysates of the P3F-C2C12 cells, it could not be identified in the exosome lysate (Fig. 1d), which agrees with our prior finding that the PAX3-FOXO1 protein is not incorporated in exosomes of human alveolar (PAX3-FOXO1 positive) RMS cells^24^. Of note, we detected a decrease in total amount of protein extracted from exosomes per million cultured cells upon expression of *PAX3-FOXO1* (Fig. 1e).

### Exosomes from *PAX3-FOXO1*-transduced C2C12 cells promote proliferation, migration and invasion of recipient cells

**Figure 2:**
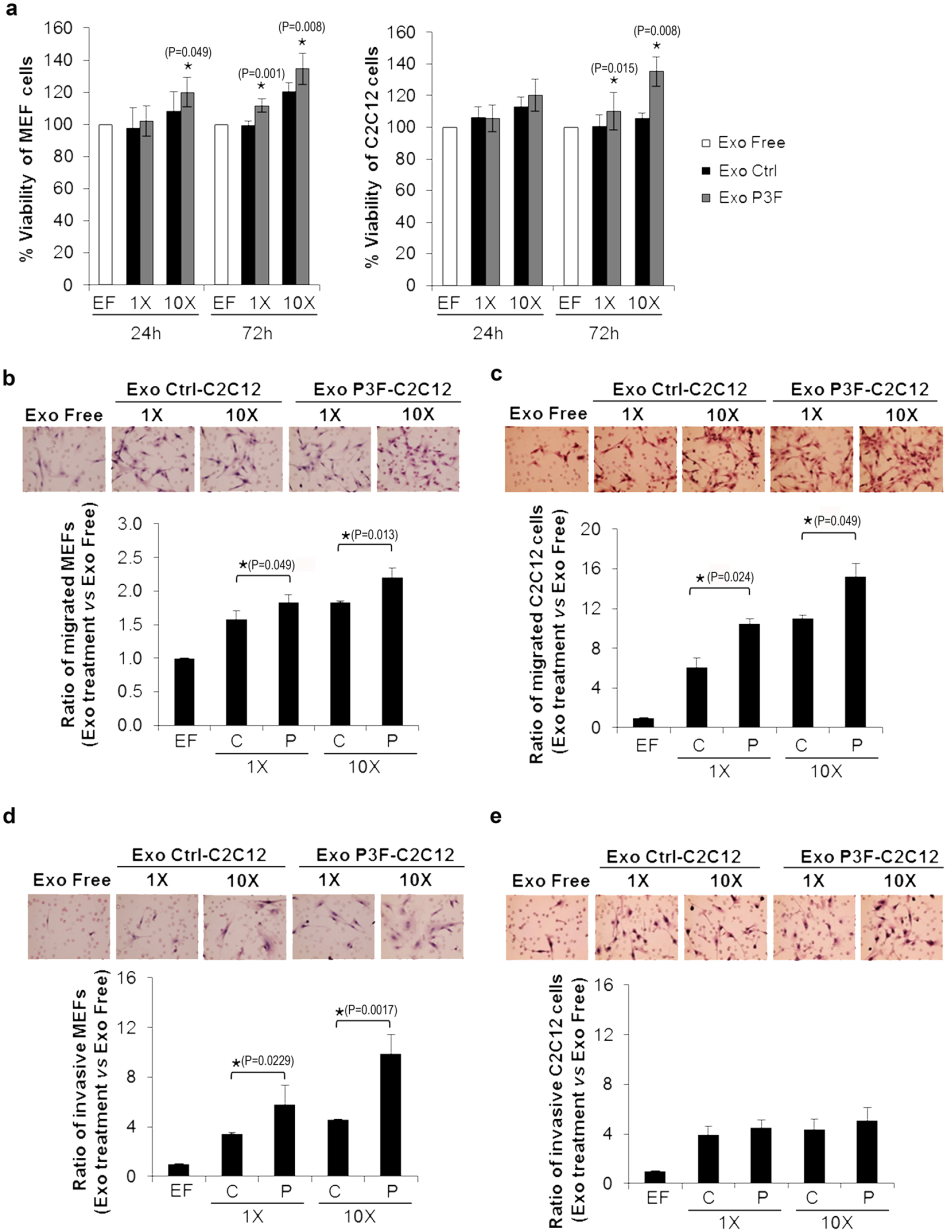
P3F-C2C12-derived exosomes promote proliferation, migration and invasion of recipient cells. (**a**) MTT assay performed on MEFs (left panel) or C2C12 cells (right panel) treated with the indicated amount of exosomes (Exo) for 24 or 72 hours, as indicated. Control condition is cells treated with exosome-free media. (**b**–**e**) Representative photomicrographs for transwell migration assay of MEFs (**b**) and C2C12 cells (**c**), and transwell invasion assay of MEFs (**d**) and C2C12 (**e**) treated with specified amount of exosomes (1X and 10X) for 24 hours, compared to control (treated with exosome-free media) cells. Histograms represent quantitation of the cell ratio versus control at the denoted conditions. Bars represent standard deviation. Asterisks denote a statistically significant difference (p-value < 0.05).

We used isolated exosomes from P3F-C2C12 and Ctrl-C2C12 to treat normal mouse embryonic fibroblasts (MEFs) and non-transduced C2C12 cells. Using MTT viability assay, we found that MEFs treated with P3F-C2C12 exosomes, but not Ctrl-C2C12 exosomes, showed increase in proliferation by 72 hours after treatment, at both 1X exosome concentration (amount of exosomes isolated from similar number of cells to treated cells), or 10X exosomes (10 times that amount) (Fig. 2a, left panel). The same effect was observed when C2C12 cells were used as recipient cells (Fig. 2a, right panel). Transwell migration assay showed that, while both P3F-C2C12 exosomes and Ctrl-C2C12 exosomes led to an increase in migration ability of MEFs, P3F-exosome treated cells had significantly higher numbers of migrated cells, at both 1X and 10X exosome concentrations (Fig. 2b). Similar results were noted when C2C12 cells were used as the recipient cells (Fig. 2c). Matrigel transwell invasion assay revealed that MEFs treated with P3F-C2C12 derived exosomes had much higher numbers of invading cells than those treated with Ctrl-C2C12 derived exosomes, at both 1X and 10X concentrations (Fig. 2d). However, when C2C12 cells were used as recipient cells, the number of invading cells was similar irrespective of amount (1X or 10X) or nature (P3F-C2C12 or Ctrl-C2C12 derived) of exosomes (Fig. 2e). Thus, we conclude that *PAX3-FOXO1* modulates exosomes of myoblasts, with a resultant increase in proliferation, migration, and invasion of recipient fibroblasts, as well as increased proliferation and migration of recipient myoblasts.

### *PAX3-FOXO1* alters the miRNA content of exosomes

**Figure 3:**
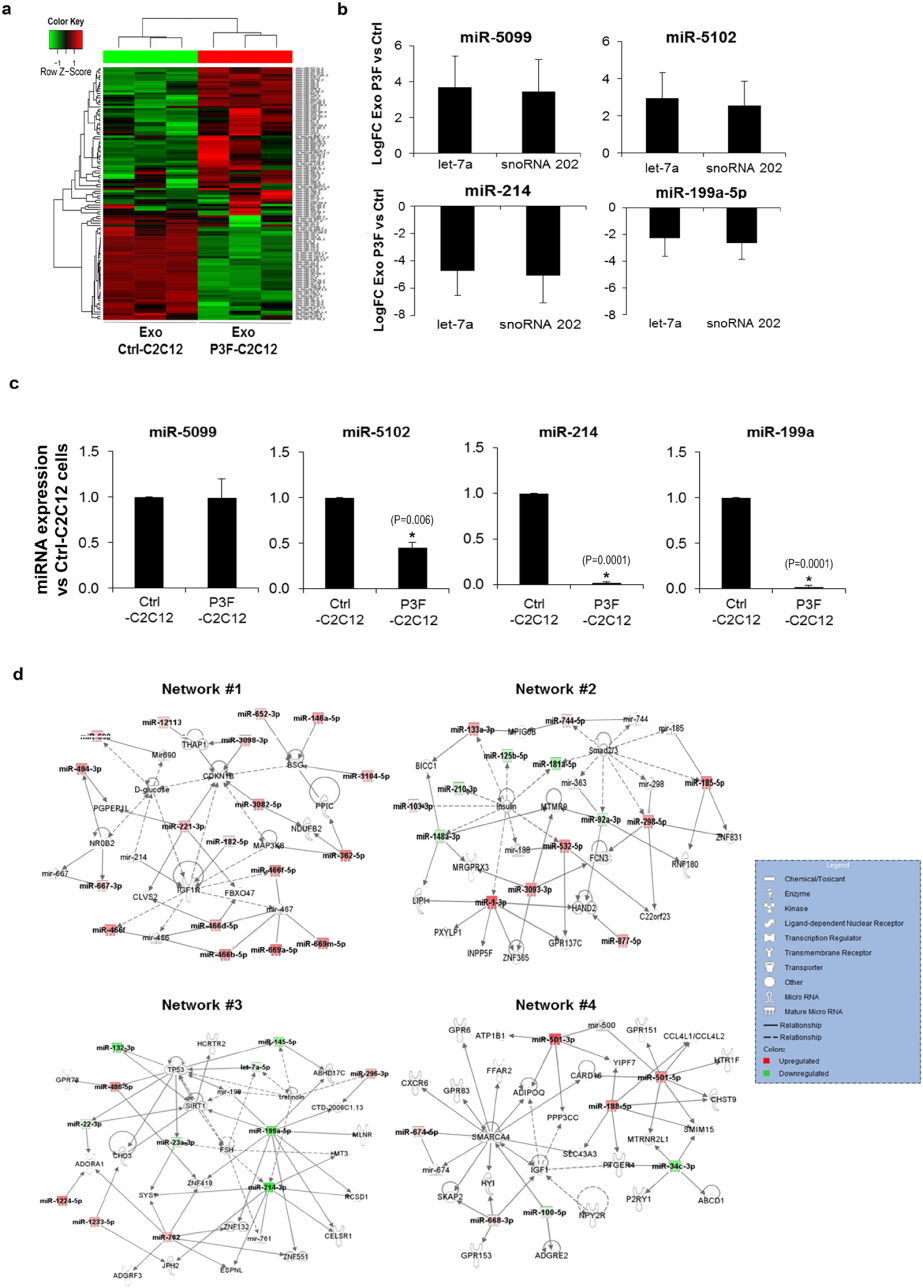
PAX3-FOXO1 fusion oncoprotein alters the miRNA content of exosomes. (**a**) Hierarchical clustering of the deregulated miRNA P3F-C2C12 derived exosomes compared to Ctrl-C2C12 derived exosomes. Each column represents an exosome isolated replicate, and each row represents a miRNA. The scaled expression of each miRNA, denoted as the row Z-score, is plotted in green–red color scale. High expression levels are indicated in green and low expression levels are shown in red. (**b**) Histograms representing mean log fold change (logFC) of the indicated miRNA in P3F-C2C12 versus Ctrl-C2C12 derived exosomes. Results are shown using two different endogenous controls (Let-7a and snoRNA202), as indicated. Values are means of 3 independent exosome preparations, each run in duplicates. Bars represent standard deviation. (**c**) Histograms representing indicated miRNA levels in P3F-C2C12 cells versus Ctrl-C2C12 cells, normalized to snoRNA202 expression detected by qRT-PCR analysis. Values presented are means of three independent experiments. Bars represent standard deviation. Asterisks denote a statistically significant difference (p-value < 0.05). (**d**) IPA Network analysis of deregulated exosomal miRNA due to PAX3-FOXO1 transduction in C2C12 cells reveals 4 networks that contain more than one identified focus miRNA. These networks were generated through the use of IPA (QIAGEN Inc., https://www.qiagenbioinformatics.com/products/ingenuity-pathway-analysis).

To analyze the effect of *PAX3-FOXO1* on exosome cargo, we focused on miRNA content, as our previous work had shown that small RNA accounted for the major proportion of exosome RNA^24^. Unsupervised hierarchal clustering of miRNA microarray profiling showed that miRNA of P3F-C2C12 derived exosomes clustered together, and clearly separated from miRNA of Ctrl-C2C12 derived exosomes (Fig. 3a). There were 91 enriched and 20 depleted miRNA, as listed in Supporting Information: Tables S1 and S2, respectively. Using quantitative RT-PCR, we used 2 internal controls, *let7a* and *snoRNA 202*, to verify a subset of the identified enriched and depleted miRNA, including the enriched *miR-5099*, *miR-5102*, and the depleted *miR-214* and *miR199a-5p*, showing excellent concordance with the microarray data (Fig. 3b). Interestingly, while *miR-5099* and *miR-5102* were highly enriched in P3F isolated exosomes compared to Ctrl exosomes, they were not enriched in the corresponding P3F-C2C12 cells compared to Ctrl-C2C12 cells (Fig. 3c). On the other hand, *miR-214* and *miR-199a* showed similar differential expression in exosomes as in corresponding cells. Thus, it seems that the differential expression of miRNA in exosomes can be reflective of their enrichment in cells, whereas others are selectively regulated within exosomes irrespective of relative levels within the parental cells.

Ingenuity Pathway Analysis (IPA) identified the deregulated miRNA as primarily involved in cancer and inflammation signaling pathways (Supplementary Table S3), underscoring a possible role in invasion and metastasis. Network prediction using IPA identified 4 putative networks with more than one focus molecule (Fig. 3d). Network 1 included transcriptional regulators such as NR0B2, molecules involved in energy metabolism such as D-glucose, the receptor tyrosine kinase IGF1R known to be important in RMS biology^25^, and the tumor suppressor protein CDKN1B, among others. Network 2 included transcriptional modulators and intracellular signaling molecules implicated in tumorigenesis such as SMAD2/3, as well as proteins involved in metabolic pathways such as RNF180 and Insulin. Network 3 included the tumor suppressor TP53, and SIRT1 previously implicated in tumorigenesis^26^. Network 4 included chromatin-remodeling proteins such as SMARCA4 (which is a tumor suppressor protein)^27^, and the growth signaling factor IGF1 known to be implicated in RMS^28^.

### *miR-486-5p* is a downstream effector of *PAX3-FOXO1* in exosome-mediated paracrine signaling

Of the enriched exosomal miRNA (Supporting Information Table S1), we focused on *miR-486-5p*, because its role has been reported as oncogenic or tumor suppressive depending on cell type and context, including a recent report in RMS^18,29–31^. Mechanistically, *miR-486-5p* has been shown to be involved in different pathways targeting key proteins such as PTEN^32,33^, PIK3R1^34^, NF-κB-negative regulators such as CYLD and Cezanne^35^, and FOXO1^32^.

**Figure 4:**
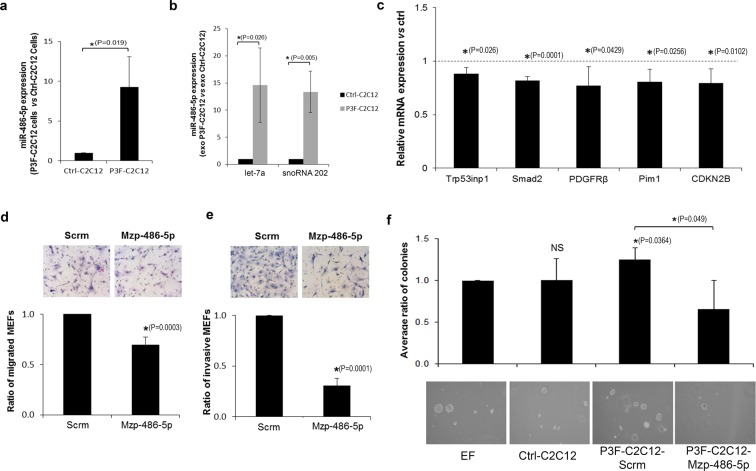
*miR-486-5p* is a downstream effector of PAX3-FOXO1 exosome-mediated effects. (**a**,**b**) qRT-PCR analysis of *miR-486-5p* expression in (**a**) P3F-C2C12 cells and (**b**) their derived exosomes, relative to Ctrl-C2C12 cells and their derived exosomes, respectively, using 2 distinct internal controls, *let-7a* and *snoRNA202* (**c**) Relative expression of miR-486-5p known targets that were significantly downregulated in MEFs treated for 48 h with P3F-C2C12 exosomes normalized to those treated with Ctrl-C2C12 exosomes as detected by qRT-PCR.GAPDH was used as internal control. (**d**,**e**) Migration (**d**) and invasion (**e**) of MEFs treated with 10X exosomes extracted from P3F-C2C12 cells transduced either with negative control (*Scrm*), or with knockdown of *miR-486-5p* by *MiRZip-486* (*Mzp-486-5p*), as indicated. Histograms represent ratio of the denoted conditions *versus* control. (**f**) Ratio of number of colonies formed by P3F-C2C12 cells after treatment with the specified exosomes at 10X, to that of cells treated with control exosome-free media. Representative microscope images are of colonies at 3 weeks. Values are mean ratio of three independent experiments. Bars represent standard deviation. Asterisks denote a statistically significant difference (p-value < 0.05).

To study the role of *miR-486-5p* in PAX3-FOXO1 mediated effects of exosomes, we verified that *miR-486-5p* was increased in exosomes in response to *PAX3-FOXO1* expression, using 2 different internal controls (Fig. 4a). In addition, qRT-PCR showed an approximately four-fold log increase of *miR-486-5p* in P3F-C2C12 cell lysates, as compared to that of Ctrl-C2C12 cells (Fig. 4b). To identify whether downstream targets of *miR-486-5p* were indeed modulated in recipient cells, we interrogated a panel of 13 previously validated targets of *miR-486-5p*^18,36–40^, all of which have been implicated in oncogenesis. We found that 5 of those targets: *Smad2*, *Trp53inp1*, *Pdgfrb*, *Pim1*, and *Cdkn2b* were significantly down-regulated in fibroblasts treated with P3F-C2C12 derived exosomes *vs*. those treated with control exosomes (Fig. 4c), whereas the remaining targets did not show a significant change (Supporting Information: Figure S1a), suggesting differential pathway modulation in this setting.

To evaluate whether *miR-486-5p* is involved in mediating the effects of P3F*-*C2C12 exosomes on recipient cells, we down-regulated *miR-486-5p* in P3F-C2C12 cells, and collected the exosomes. We used the *MZIP486-5p-PA-1* construct, which is a lentivirus vector expressing a commercially available short hairpin RNA (shRNA) leading to *miR-486* inhibition by being zipped to an anti-sense inhibitor. *MZIP486-5p-*transduction of P3F-C2C12 cells reversed the effects of their derived exosomes on both recipient fibroblast migration and invasion (Fig. 4d,e), and on colony formation capabilities of recipient P3F-C2C12 cells (Fig. 4f). The results were similar when *miR-486-5p* knockdown was performed using an oligonucleotide inhibitor instead of *MZIP486-5p* construct (Supporting Information: Figure S1b–d).

**Figure 5:**
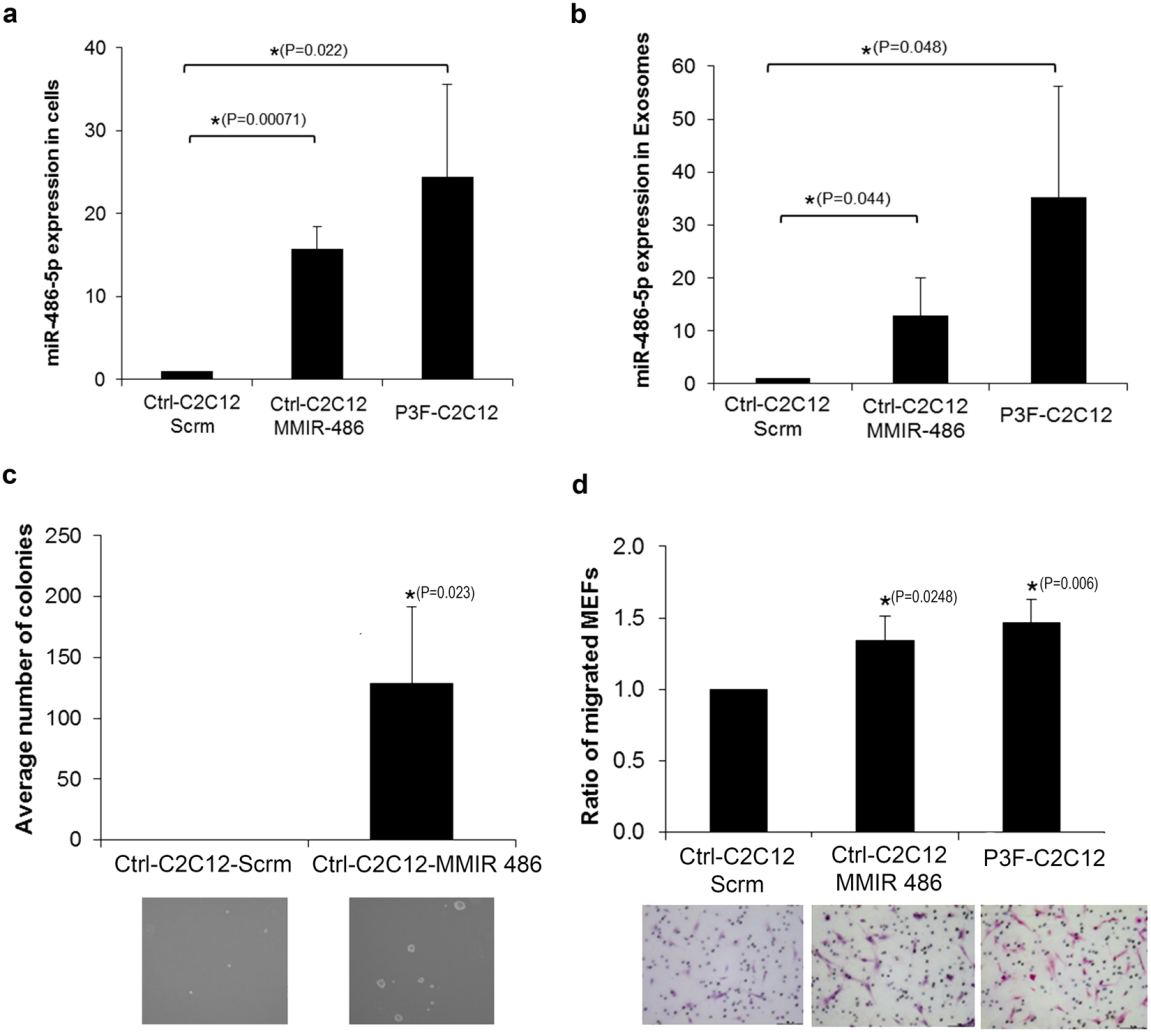
Overexpressing miR-486-5p in C2C12 cells leads to exosome effects mimicking those of PAX3-FOXO1 expression. (**a**,**b**) qRT-PCR analysis of miR-486-5p expression in (**a**) C2C12 cells and (**b**) their derived exosomes, after transduction with either negative control vector (Scrm) or miR-486-5p overexpressing vector (MMIR-486). Values are normalized to Ctrl-C2C12-Scrm cells and their derived exosomes. (**c**) Colony formation assay of C2C12 cells transduced with either Scrm or MMIR-486, as indicated. (**d**) Ratio of migratory MEFs after treatment with the specified exosomes at 10X for 24 hours. Values presented are means of three independent experiments. Bars represent standard deviation. Asterisks denote a statistically significant difference (p-value < 0.05).

To further evaluate whether increased *miR-486-5p* expression can phenocopy effects of *PAX3-FOXO1* on exosome function, we constitutively expressed *miR-486-5p* in C2C12 cells using a retroviral expression vector *MMIR-486-5p*. This resulted in a fifteen-fold increase in *miR-486-5p* levels, closely approximating the levels noted in P3F-C2C12 cells (Fig. 5a). Importantly, this resulted also in an increase in exosome content of *miR-486-5p*, again to a level approximating that found in P3F-C2C12-derived exosomes (Fig. 5b). Interestingly, overexpressing *miR-486-5p* in Ctrl-C2C12 cells induced anchorage independent growth mimicking those of P3F expression (Fig. 5c). Similarly, treatment of fibroblasts with exosomes derived from *miR-486-5p*-expressing C2C12 cells (*MMIR-486-*C2C12) resulted in increased migration, similar to that seen with exosomes derived from P3F-C2C12 cells (Fig. 5d).

### *miR-486-5p* is overexpressed in cells and exosomes of human *PAX3-FOXO1*-positive rhabdomyosarcoma cells, and can be detected in serum-derived exosomes of patients with rhabdomyosarcoma

**Figure 6:**
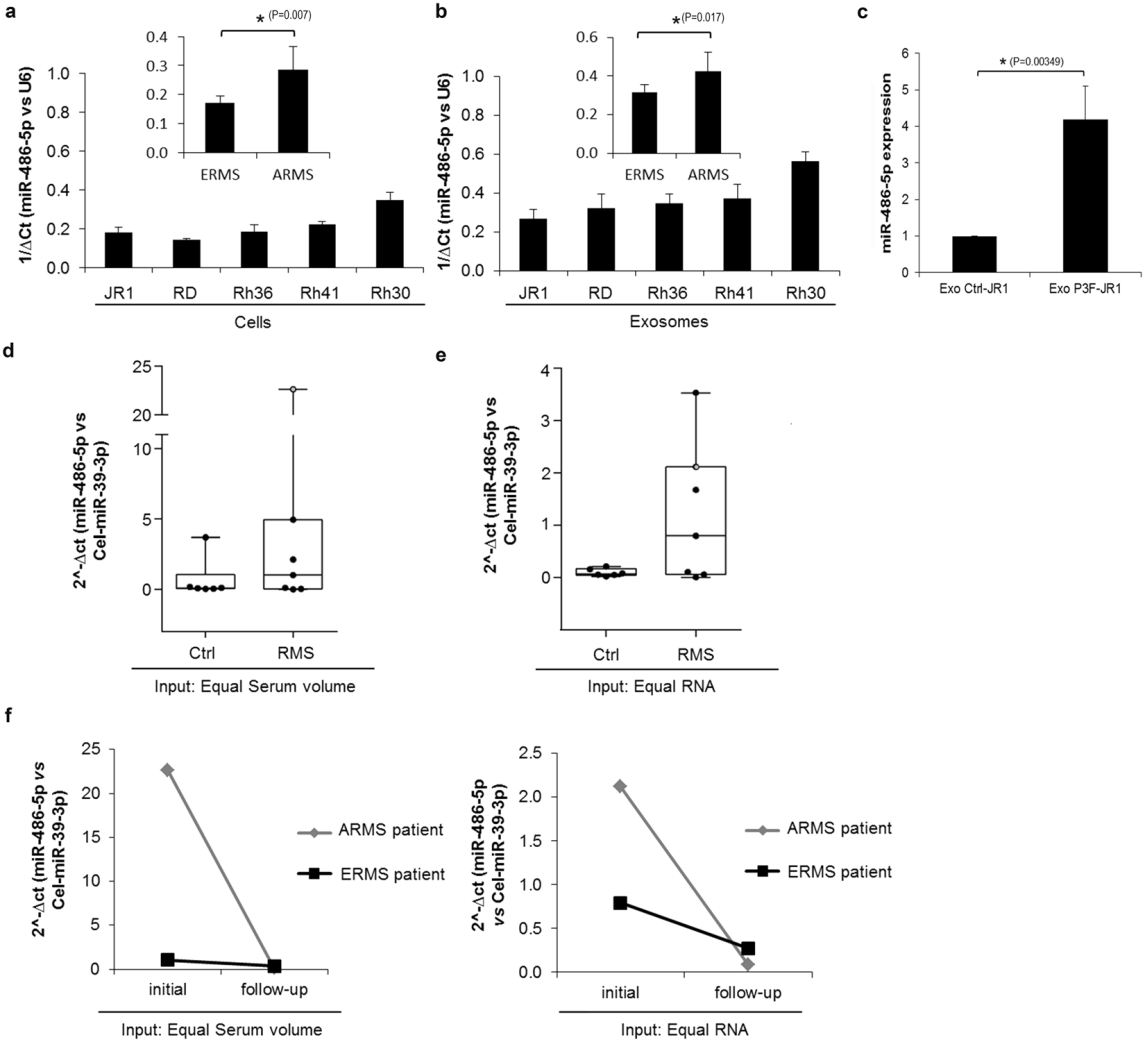
*miR-486-5p* expression in human RMS cells and patient serum samples. (**a**,**b**) qRT-PCR of *miR-486-5p* expression in (**a**) human RMS cell lines and (**b**) their derived exosomes, normalized to *U6* expression. (**c**) *miR-486-5p* levels in exosomes isolated from P3F-JR1 cells *versus* those from Ctrl-JR1 cells, normalized to *U6* expression as detected by qRT-PCR analysis. Values presented are means of three independent experiments. Bars represent standard deviation. Asterisks denote a statistically significant difference. (**d**–**e**), qRT-PCR analysis of *miR-486-5p* in exosomes isolated from the serum of 7 patients with RMS (6 ERMS and 1 ARMS labeled in grey) compared to those from control subjects with benign tumors (n = 6) starting with either (**d**) equal serum (*p-value* = *0.51*) or (**e**) equal RNA amounts (*p-value* = *0.18*). *C. elegans miR-39-3p* spike-in control was used for normalization (**f**) Exosomal *miR-486-5p* levels in follow-up serum of ARMS and ERMS patients compared to initial serum collected at diagnosis. Equal serum volume (left panel) or equal RNA (right panel) was used as input, and *C. elegans miR-39-3p* spike-in control was used for normalization.

To identify whether *miR-486-5*p is expressed in RMS cells, we examined a panel of five RMS cell lines, two of which (Rh30 and Rh41) are of the alveolar subtype and express the PAX3-FOXO1 fusion protein. We found that *miR-486-5p* was expressed in cell lysates (Fig. 6a) and exosomes (Fig. 6b) of all five RMS cell lines, but to significantly higher levels in the PAX3-FOXO1 -positive alveolar Rh30 and Rh41. Of note, expressing PAX3-FOXO1 in the ERMS cell line JR1 resulted in a 2-log increase in *miR-486-5p* levels, further confirming it as a downstream effector (Fig. 6c).

To investigate whether *miR-486-5p* was relevant to clinical settings, we extracted exosomes from equal volumes of serum from seven patients with newly diagnosed RMS (6 with embryonal RMS and 1 with alveolar RMS), as well as six age-matched control patients who had benign tumors. Although number of samples was small, *miR-486-5p* level seemed to be higher in serum–derived exosomes of RMS patients, and was highest in the one patient with alveolar (PAX3-FOXO1-positive) RMS (Fig. 6d). This analysis was similar when comparing equal amounts of RNA, rather than equal volumes of serum (Fig. 6e). Follow-up serum after treatment were available for 2 patients including the patient with ARMS, and showed a major decrease in exosome-derived *miR-486-5p* in this patient, correlating with tumor response (Fig. 6f).

## Discussion

RMS driven by the fusion protein PAX3-FOXO1, or less commonly PAX7-FOXO1, is well established to be associated with a worse prognosis as compared to fusion-negative RMS tumors, due to aggressive tumor behavior and higher risk of recurrence and dissemination^2^. Current clinical risk group stratification is moving towards classifying tumors as fusion gene-positive and fusion gene-negative, further attesting to the importance of this oncoprotein in dictating clinical tumor behavior^41^. Despite decades of studying downstream signaling pathways, it has remained difficult to pinpoint exact relevant and targetable mechanism by which its oncogenic effects are mediated, and translate those into clinical advances^12^.

Previous work has shown that PAX3-FOXO1 leads to increased proliferation of C2C12 myoblasts, with enhanced ability to form tumors in nude mice^42^. Investigated downstream mechanisms include transcriptional downregulation of Pten^43^, upregulation of Met^44^, and suppression of myogenic gene transcription^45^, among other intracellular signaling pathways affecting proliferation, cell survival, differentiation, epigenetic regulation, and metabolism (reviewed in^12^). Importantly, while multiple studies have focused on PAX3-FOXO1 in autocrine signaling, there have been no investigations of its possible paracrine effects on neighboring stromal cells. This is despite sizeable evidence from studies done in other tumor types, showing that tumor stroma fibroblasts can be altered by paracrine signaling, and may play a major role in modulating and facilitating cancer cell invasion and metastasis^46,47^.

Paracrine cellular signaling between tumor cells and associated fibroblasts can occur *via* multiple mechanisms, including secreted vesicles such as exosomes (reviewed in^23,48^). Specifically, miRNA delivered by exosomes stimulate activation of cancer-associated stromal fibroblast, leading to tumor growth and metastasis (reviewed in^29^). Our prior work has shown exosomes to be relevant mediators of paracrine effects of human RMS cells, in both fusion-positive and fusion-negative cell lines^24^. We have now uncovered an important role for *PAX3-FOXO1* in modulating exosome content of myoblasts, which resulted in pro-tumorigenic effects in recipient cells, including increased proliferation, migration, invasion, and colony formation. Thus, this effect of *PAX3-FOXO1* directly influencing paracrine signaling may help explain the well-recognized tendency for fusion protein-positive RMS to invade local structures and lead to early metastasis. *PAX3-FOXO1* altered exosome content of miRNA, with expected influenced networks centering on cancer and inflammation pathways, and proteins well recognized to play a role in RMS tumor biology, including IGF1 and IGF1R, CDKN1B, SMAD 2/3, SIRT1, TP53, and the epigenetic regulator SMARCA4. However, the specific role of each of these putative targets still needs to be confirmed and further evaluated.

We identified exosomal *miR-486-5p* as a mediator of *PAX3-FOXO1* paracrine effects, including increasing recipient cell migration, invasion, and colony formation. During the execution of this work, another study was published showing that *miR-486-5p*, among other miRNA, is a downstream effector of *PAX3-FOXO1* in RMS cells^18^. They showed that PAX3-FOXO1 directly activates transcription of *miR-486-5p* through its binding to the upstream *sANK1* promoter, and that *miR-486-5p* cellular expression leads to increased proliferation and enhancement of invasion and clonogenic growth of human RMS cell lines^18^, findings that we have now independently validated in C2C12 myoblasts as well. In addition to those effects within the cell, we now describe the novel role of both *PAX3-FOXO1* and its downstream effector *miR-486-5p* on exosome-mediated oncogenic paracrine effects. Of note, *Hanna et al*. showed that knockdown of *miR-486-5p* in fusion-positive RMS xenografts led to significant inhibition of tumor growth *in vivo*^18^. However, the extent of effect of cellular *vs*. paracrine factors *in vivo* is unclear at this stage and deserves further elucidation.

*miR-486-5p* has been described as an oncomir^29,30,35^ or as a tumor suppressor^31,38,49,50^, depending on cellular context and tumor cell type. In RMS, it seems that this miRNA acts as a potent oncogenic stimulant, as shown by our study and by Hanna *et al*.^18^. Relevant downstream targets of *miR-486-5p* responsible for its cellular effects also differ in different contexts, and that may explain its divergent function. For example, while the PI3K/AKT pathway is modulated by *miR-486-5p* in multiple cancer types such as cervical and prostate cancer and normal hematopoeitic cells^29,33^, it is not impacted in RMS cells^18^. The study by Hanna *et al*. interrogated several other known and putative targets of *miR-486-5p*, but could not identify a specific single effector protein or pathway in RMS cells, but rather subtle alterations in levels of multiple targets. Our pilot analysis of a set of downstream targets of *miR486-5p* in recipient fibroblast cells identified potential candidates of its paracrine effects, including *Trp53inp1*, *Smad2*, *Cdkn2b*, *Pdgfrβ*, and *Pim1*. Current work is ongoing to investigate the most relevant downstream effectors in this setting, for possible therapeutic targeting in this aggressive disease.

Importantly, our pilot analysis of a small number of serum samples from patients with RMS showed a tendency towards higher levels of *miR-486-5p* in exosomes of patients with RMS, and very high levels in the one patient with fusion-positive alveolar RMS. This level decreased after chemotherapy and when the patient was in remission. While this is only in one patient with ARMS, and total number of patients is small, these findings are highly suggestive of a relevant role for exosomal *miR-486-5p* in children with RMS, and warrants further investigation in a larger number of clinical samples. Positive results would suggest the use of *miR-486-5p* as a potential serum exosome biomarker for fusion-positive RMS, to aid in diagnosis, assessment of response, and follow-up of patients after treatment.

## Materials and Methods

### Cell culture

C2C12, HEK293T and RD cell lines were purchased from ATCC (Manassas, VA). JR1, Rh36, Rh30 and Rh41 cell lines were generously donated by Dr. Peter Houghton (Columbus, OH, USA). Mouse embryonic fibroblasts (MEFs) were isolated from E13.5 embryos of mixed C57BL/6 × 129/Sv 77 background (Jackson Laboratory, Maine) using the procedure approved by the Institutional Care and Use Committee (IACUC) at the American University of Beirut, and following the IACUC-approved guidelines. C2C12 cells were maintained in Dulbecco’s Modified Eagles Medium (DMEM) with 20% FBS, 1% glutamine, and 1% Pen/Strep (Sigma). Other cells were cultured in RPMI-1640 medium with 10% fetal bovine serum, 1% glutamine, and 1% Pen/Strep (Sigma). All cells maintained under standard conditions (humidified atmosphere, 95% air, 5% CO_2_, 37 °C).

### Plasmids, virus production and cell transduction

MSCV-IRES-GFP-Pax3-FOXO1 (MSCV-P3F) and MSCV-IRES-GFP (MSCV-GFP) plasmids were a kind gift from Dr. Gerard Grosveld (St. Jude Children’s Research Hospital, Memphis). MiRZip-486-5p (MZIP486-5p-PA-1), MiRzip-scrm (MZIP000-PA-1 pGreenPuro Scramble Hairpin Control), MMIR-486 (MMIR-486-PA-1-microRNA Expression Construct) and MMIR-scrm (MMIR-000-PA-1 Mouse precursor Scramble negative control) were purchased from System Biosciences (USA). 293T were transfected with MSCV-P3F or MSCV-GFP using calcium phosphate. Viral supernatants were harvested at 48 h and 72 h. Virus particles were packaged using pPACKH1 HIV Lentivector Kit-LV500A-1 (System Biosciences, USA). Cells were transduced in suspension at 32 °C, 1250 × g for 1 h with 8 μg/ml Polybrene (hexadimethrine bromide; Sigma), and sorted using FACS Aria SORP cell sorter (BD) after selection with 2 μg/ml Puromycin (Abcam). For miRNA silencing, cells were transfected with 40 pmol of anti-mir-486-5p inhibitor (GenePharma, China) using Lipofectamine RNAiMAX (Invitrogen).

### Exosome isolation

Exosomes isolation was by ultracentrifugation as described previously^24^. Cells were treated with exosomes at 1X and 10X concentration, where 1X corresponds to exosomes isolated from an equivalent number of cells to those treated. Patient serum was obtained from a clinical biorepository at the American University of Beirut Medical Center (AUBMC), governed by the institutional review board (IRB) for research use. 400 μl serum samples were thawed and diluted with PBS, and exosomes isolated using sequential centrifugation followed by ExoQuick (EXOQ5A-1, System Biosciences) extraction. Exosome morphology was examined using Scanning Electron Microscopy (SEM). Pellets were fixed in 2% paraformaldehyde and 1% glutaraldehyde (Sigma). The sample was applied to a continuous carbon grid, washed in distilled H2O, then dehydrated, left to dry, and observed using a Zeiss SEM at 30 kV. ImageJ software was used for analysis.

### RNA extraction, miRNA profiling and analysis

Cells and exosomes were lysed using QIAzol Lysis reagent (Qiagen). Total RNA was extracted using Phenol-chloroform. For human serum, 10 fmol of synthetic Caenorhabditis elegans miR (cel-miR-39, Invitrogen) was added into the denaturing solution for normalization before RNA extraction. RNA was quantified with ND-1000 spectrometer (Nanodrop Technologies, Wilmington, DE, USA), and quality assessed using the Experion electrophoresis system via standard RNA chips (Bio-Rad). MiRNA profiling was done as previously described^24^ using Affymetrix GeneChip miRNA 3.0 Arrays kit. The arrays were washed and stained on the Affymetrix Fluidics station 450. Scanning was performed using the Affymetrix gene chip scanner 3000 7 G (Affymetrix, Santa Clara, CA, USA) followed by analysis by the R statistical environment. Normalization was done using *rma* algorithm^51^, annotations derived using *biomaRt*^52^, differential expression measured using *LIMMA*7^53^ and visualization for differentially expressed miRNAs using *gplots*. A 1.5-fold change and a false discovery rate (FDR) < 0.05 were used as cut-off. Data were deposited in ArrayExpress database (www.ebi.ac.uk/arrayexpress) under accession number E-MTAB-7646. Data were analyzed through the use of IPA (QIAGEN Inc., https://www.qiagenbioinformatics.com/products/ingenuitypathway-analysis)^54^. Statistical significance (p < 0.05) was determined using Fisher’s exact test.

### Quantitative real-time polymerase chain reaction

For miRNA expression, qPCR was performed using TaqMan miRNA Assays (Applied Biosystems): mmu-miR-5099, mmu-miR-5102, hsa-miR-486, hsa-miR-214, hsa-199a-5p and cel-miR-39. The small nuclear U6 RNA (RNU6) was used as endogenous control for human samples, and hsa-let-7a and snoRNA202 for mouse samples. cDNA was synthesized using TaqMan microRNA Reverse Transcription Kit (Applied Biosystems). Real-time PCR was performed with TaqMan probes on a CFX96 real-time PCR detection system (Bio-Rad) as follows: 10 min at 95 °C, 40 cycles of 95 °C for 15 s and 60 °C for 1 min. Relative miRNA expression levels were compared using 2^−ΔΔCt^ method. Quantitative miRNA data were analyzed using CFX96 real-time PCR detection system (Bio-Rad).

For mRNA expression of potential miR-486 targets, MEFs treated with 10X P3F-C2C12 exosomes or Ctrl-C2C12 exosomes for 48 h were lysed and total RNA was reverse transcribed using QuantiTect Reverse Transcription kit (Qiagen) followed by qPCR with QuantiFast SYBR Green PCR (Qiagen) for 40 cycles using primers listed in supporting information Table S4. GAPDH was used as an internal control. Experiments were done in triplicates, and data analysis was performed using the ΔΔCT method.

### Western blot

Cells and exosomes were lysed using CHAPS lysis buffer mixed with 25X protease inhibitor (Roche), and sonication for 15 cycles. Protein concentrations were determined by Bradford method (Bio-Rad). Equal amounts of proteins were loaded and separated using 10% SDS-PAGE, transferred to polyvinylidene difluoride (PVDF) membranes (Bio-Rad) and incubated with primary antibodies: anti-HSC70, anti-GAPDH, anti-Calnexin (Santa Cruz Biotechnology), anti-TSG101 (Abcam), anti-FOXO-1 (Cell Signaling), then incubated with species-specific HRP-conjugated secondary antibodies (Santa Cruz), and detected using ECL reagent (Bio-Rad).

### *In vitro* assays

Cell Viability was quantified using MTT Kit (Roche). Cells were grown in 96-well plates then treated with specified exosomes in exosome-free media. Optical density was measured at 595 nm on a microplate reader. For migration and invasion assays, cells were added in exosome-free media onto the top chambers of 8-μm pore cell culture inserts (BD Falcon) with or without 10% growth factor reduced Matrigel (BD Biosciences). Inserts were placed in 500 μl of serum-free medium in a 24-well plate. After four hours, exosomes were added for 24hrs (migration) or 48hrs (invasion). Cells attached to the bottom membrane were fixed in 4% PFA, stained with hematoxylin and eosin, photographed using Olympus CX41 Microscope, and quantified using ImageJ software. Anchorage Independent growth was assessed by growing cells in SeaPlaque agarose (Lonza). 0.8% bottom agar was allowed to solidify and layered with 0.48% top agar mixed with 15,000 cells, in 6-well plate and 1 ml media. Media was replaced twice a week. Visible colonies were counted from at least 5 different fields at 4X magnification (ZEISS Primo Vert Microscope).

### Human samples

All experiments were performed in accordance with relevant institutional and national guidelines and regulations. All human studies were approved by the Institutional Review Board (IRB) at the American University of Beirut Medical Center (AUBMC). Informed consent for serum collection and use for research was obtained from all subjects, and from parents/legal guardians for subjects younger than 18 years of age. In addition to legal guardian consent, assent was additionally obtained from minors over 7 years of age, as per IRB regulations.

### Statistical analysis

*miR-486-5p* enrichment in serum samples was determined using nonparametric Mann-Whitney test. Statistical significance was set as p < 0.05. Statistical analysis was conducted using Prism Software (GraphPAd 6.01, La Jolla, CA, USA.)

## Supplementary information

Supplementary Information
