# Supplementary material for: The PAX3-FOXO1 oncogene alters exosome miRNA content and leads to paracrine effects mediated by exosomal miR-486
Source: Sci Rep. 2019 Oct 2;9:14242. doi: 10.1038/s41598-019-50592-4 (PMC6775163; doi:10.1038/s41598-019-50592-4)
Supplement: Supplementary file 1 — Supplementary Information [file 41598_2019_50592_MOESM1_ESM.pdf]

The *PAX3-FOXO1* oncogene alters exosome miRNA content and leads to paracrine effects mediated by exosomal *miR-486*

Farah Ghamloush [1], Sandra Ghayad [2], Ghina Rammal [2], Assil Fahs [2], Abeer J. Ayoub [2], Zeina Merabi [1], Mohamad Harajly [1], Hassan Zalzali [1], Raya Saab [1,3]*

[1] Department of Pediatrics and Adolescent Medicine, Children's Cancer Institute, American University of Beirut, Beirut, Lebanon.

[2] Department of Biology, Faculty of Science II, Lebanese University, Fanar, Lebanon.

[3] Department of Anatomy, Cell Biology and Physiology, American University of Beirut, Beirut, Lebanon

***Correspondence to:** Raya Saab, MD, Department of Pediatrics and Adolescent Medicine, Children's Cancer Institute, American University of Beirut Medical Center, Riad El Solh Street, Beirut 1107 2020, Lebanon. Tel.: +961-1-350000. Email: rs88@aub.edu.lb

Supplementary Figure S1:

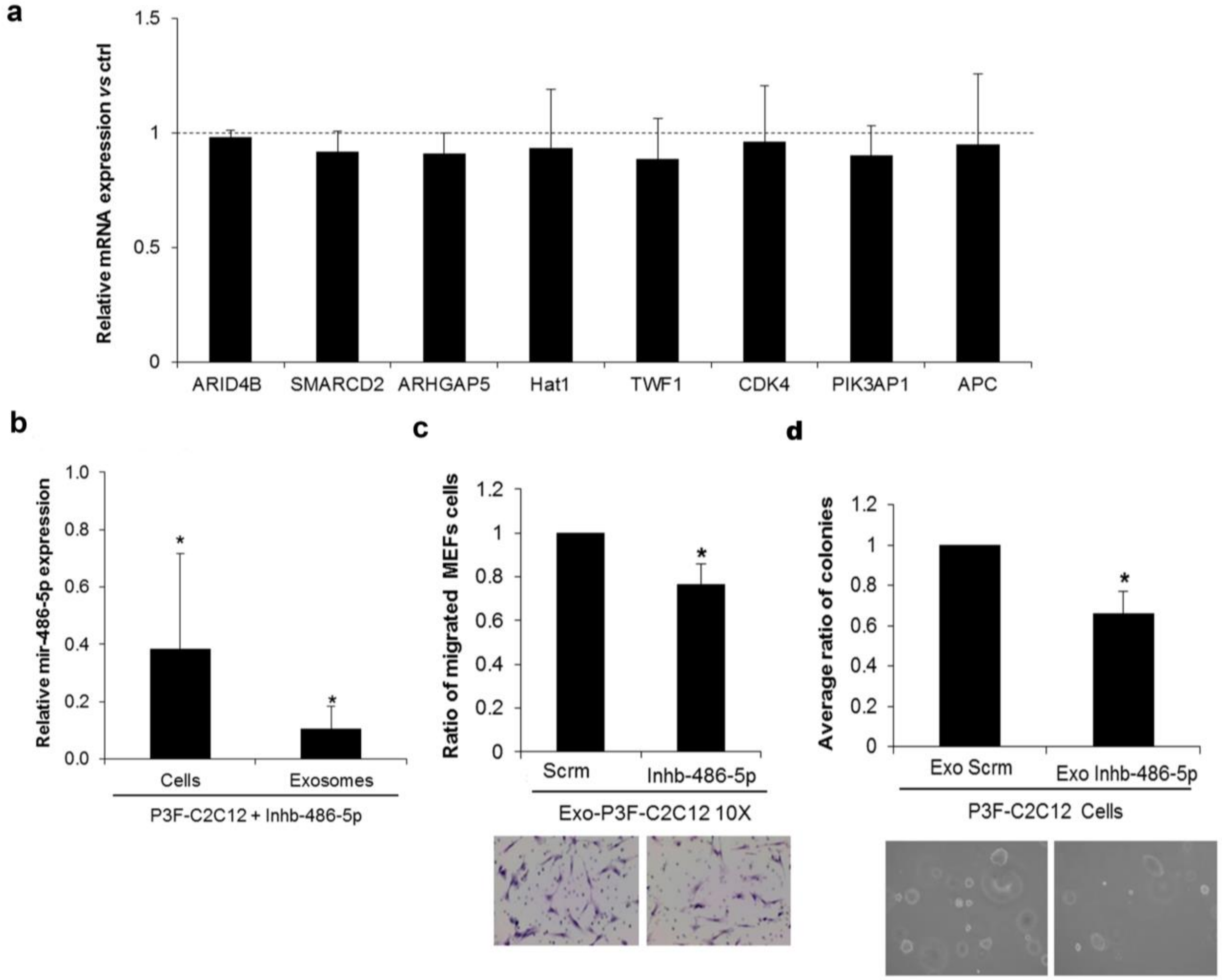

Figure S1: (a) Negative qRT-PCR results for an additional panel of 8 previously validated miR-486 downstream targets, in MEFs treated for 48h with P3F-C2C12 exosomes normalized to those treated with Ctrl-C2C12 exosomes. GAPDH was used as internal control. (b) Relative *miR-486-5p* expression in cells and exosomes of P3F-C2C12 cells transiently transfected with 40 *pmol* of either *miR-486-5p* inhibitor (*Inhb-486*) *vs* negative control (*Scrm*) after 96 hours. (c) Ratio of migrated MEFs after treatment with 10X exosomes for 24 hours as indicated. (d) Ratio of colonies formed by P3F-C2C12 cells in soft agar treated with 10X exosomes for 3 weeks as indicated.

Values presented are means of three independent experiments and determined *versus Scrm*. Bars represent standard deviation. Asterisks denote a statistically significant difference (p-value < 0.05).

Supplementary Figure S2:

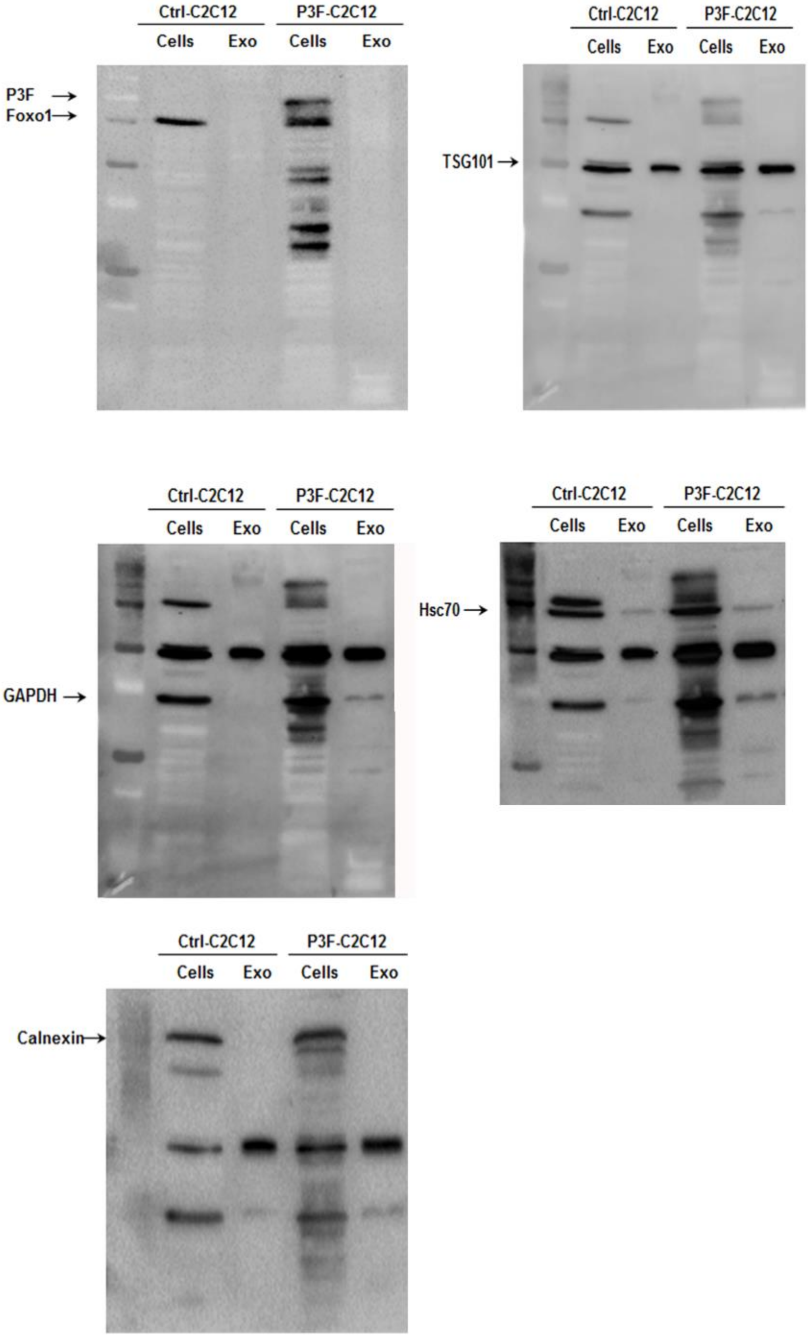

Figure S2: Full western blots of Foxo1, TSG101, GAPDH, Hsc70 and Calnexin in cells and exosomes of Ctrl-C2C12 and P3F-C2C12.

Table S1. Significantly enriched miRNAs in exosomes of P3F-expressing C2C12 cells

Enriched miRNA	logFC	adj.P.Val
mmu-miR-5099	6.480	0.003
mmu-miR-5102	5.825	0.001
mmu-miR-501-3p	5.558	0.002
mmu-miR-206	5.195	0.002
mmu-miR-677-star	5.165	0.000
mmu-miR-500	4.881	0.008
mmu-miR-92a-2-star	4.701	0.002
mmu-miR-669f-5p	4.384	0.010
mmu-miR-714	4.257	0.006
mmu-miR-362-5p	4.141	0.001
mmu-miR-1224	4.137	0.012
mmu-miR-665-star	4.111	0.015
mmu-miR-1949	3.979	0.047
mmu-miR-1249-star	3.907	0.004
hp_mmu-mir-1194	3.879	0.003
mmu-miR-1956	3.819	0.015
mmu-miR-3473	3.811	0.004
mmu-miR-669m-5p	3.764	0.010
mmu-miR-1940	3.764	0.029
mmu-miR-532-5p	3.614	0.001
mmu-miR-195-star	3.569	0.039
mmu-miR-466f-5p	3.535	0.015
mmu-miR-25-star	3.510	0.001
mmu-miR-1187	3.497	0.027
mmu-miR-1934-star	3.484	0.009
mmu-miR-466f	3.447	0.014
mmu-miR-188-5p	3.439	0.001
mmu-miR-501-5p	3.437	0.019
**mmu-miR-486-5p**	**3.363**	**0.035**
mmu-miR-3093-3p	3.359	0.001
mmu-miR-466m-5p	3.344	0.027
mmu-miR-494	3.282	0.003
mmu-miR-5132	3.194	0.035
mmu-miR-3082-5p	3.175	0.029
mmu-miR-298	3.060	0.001
hp_mmu-mir-466f-2_x	3.010	0.024
mmu-miR-146a	3.009	0.032
mmu-miR-5122	2.929	0.022
mmu-miR-1982-star	2.871	0.010
mmu-miR-3102-star	2.824	0.002
mmu-miR-133b	2.797	0.022
mmu-miR-3107	2.760	0.018

Enriched miRNA	logFC	adj.P.Val
mmu-miR-705	2.753	0.014
mmu-miR-346-star	2.719	0.014
mmu-miR-5105	2.713	0.025
mmu-miR-877	2.565	0.003
hp_mmu-mir-466f-1_x	2.554	0.035
mmu-miR-466i-5p	2.469	0.018
mmu-miR-466j	2.400	0.019
mmu-miR-3090-star	2.373	0.035
mmu-miR-296-3p	2.321	0.029
mmu-miR-1906	2.279	0.002
mmu-miR-762	2.267	0.048
hp_mmu-mir-466f-3_x	2.251	0.014
mmu-miR-3098-3p	2.225	0.004
mmu-miR-3104-5p	2.180	0.029
mmu-miR-221	2.076	0.003
mmu-miR-711	2.011	0.046
mmu-miR-690	1.958	0.038
mmu-miR-504-star	1.922	0.010
mmu-miR-3072-star	1.883	0.007
mmu-miR-652-star	1.868	0.048
mmu-miR-222	1.711	0.005
mmu-miR-183-star	1.655	0.042
mmu-miR-150-star	1.631	0.026
mmu-miR-652	1.452	0.029
mmu-miR-345-3p	1.387	0.010
hp_mmu-mir-466m_x	1.380	0.022
hp_mmu-mir-500	1.335	0.022
hp_mmu-mir-501	1.322	0.018
mmu-miR-770-5p	1.238	0.014
hp_mmu-mir-677	1.231	0.014
hp_mmu-mir-3069_x	1.214	0.034
mmu-miR-3473d	1.193	0.040
hp_mmu-mir-677_x	1.193	0.014
mmu-miR-667	1.168	0.015
mmu-miR-204-star	1.117	0.019
mmu-miR-1943-star	1.095	0.033
mmu-miR-744	1.028	0.026
mmu-miR-182	1.015	0.050
hp_mmu-mir-466j_x	1.000	0.024
mmu-miR-5126	0.989	0.035
mmu-miR-668	0.900	0.040
hp_mmu-mir-1956	0.900	0.038
hp_mmu-mir-194-2_x	0.896	0.040
mmu-miR-107	0.891	0.014

Enriched miRNA	logFC	adj.P.Val
hp_mmu-mir-5122	0.847	0.025
hp_mmu-mir-466h_x	0.821	0.035
hp_mmu-mir-3074-2	0.795	0.026
mmu-miR-221-star	0.746	0.038
hp_mmu-mir-5098_x	0.715	0.035

Table S2. Significantly depleted miRNAs in exosomes of P3F-expressing C2C12 cells

Depleted miRNA	logFC	adj.P.Val
hp_mmu-mir-140	-0.795	0.035
mmu-miR-23a	-0.893	0.010
mmu-let-7e	-0.935	0.033
mmu-miR-181b	-0.997	0.021
mmu-miR-22	-0.999	0.019
mmu-miR-92a	-1.091	0.035
mmu-miR-99b	-1.110	0.019
mmu-miR-23b	-1.363	0.035
mmu-miR-210	-1.489	0.050
mmu-miR-125a-5p	-1.502	0.003
mmu-miR-152	-1.854	0.049
mmu-miR-5100	-2.446	0.010
mmu-miR-140-star	-2.578	0.004
mmu-miR-181a	-2.616	0.014
mmu-miR-145	-2.636	0.002
mmu-miR-34b-3p	-3.622	0.021
mmu-miR-132	-4.167	0.003
mmu-miR-34c-star	-4.285	0.007
mmu-miR-199a-5p	-4.369	0.003
mmu-miR-214	-6.143	0.003

Table S3. Significantly represented diseases and functions for the deregulated miRNA using IPA.

Categories	Diseases or Functions Annotation	p-Value	Number of molecules
Organismal Injury and Abnormalities, Reproductive System Disease	Nonobstructive azoospermia	8.65E-20	14
Cancer, Organismal Injury and Abnormalities, Reproductive System Disease	Early stage invasive cervical squamous cell carcinoma	2.23E-15	9
Inflammatory Response, Organismal Injury and Abnormalities	Inflammation of organ	3.4E-15	21
Inflammatory Disease, Inflammatory Response, Organismal Injury and Abnormalities, Renal and Urological Disease	Class II lupus nephritis	5.25E-14	9
Cancer, Organismal Injury and Abnormalities	Primary melanoma	9.76E-14	8
Inflammatory Response	Inflammation of absolute anatomical region	3.87E-12	17
Cancer, Organismal Injury and Abnormalities	Early stage solid tumor	4.57E-12	10
Endocrine System Disorders, Gastrointestinal Disease, Metabolic Disease, Organismal Injury and Abnormalities	Non-insulin-dependent diabetes mellitus	1.04E-11	12
Cancer, Organismal Injury and Abnormalities, Reproductive System Disease	Cervical squamous cell carcinoma	4.63E-11	10

Table S4. Primer sequences of miR-486 downstream targets.

Gene	Primer sequence
**Trp53inp1**	F; TCCTCAGCAGAGCACACTTC<br>R: TCCATTGGACAGGACTCAAA
**Smad2**	F: AAGCCATCACCACTCAGAATTG<br>R: CACTGATCTACCGTATTTGCTGT
**ARID4B**	F: AACAAAGGTGCAGGTGAAGC<br>R: ACATCAGTGCCCACTGTCAA
**SMARCD2**	F: CTATGGGCCGGACAACCATC'<br>R: TGTGCACTCCCAGCAACCTC'
**PDGFRβ**	F: TTCCAGGAGTGATACCAGCTT<br>R: AGGGGGCGTGATGACTAGG
**Pim1**	F: ATTCCGTTTGAGCACGATGAA<br>R:TGAAGAGACAGTTTGCCTGAAGAA
**ARHGAP5**	F: TCCTGCAATCACATCTGACC<br>R: GAGGGGCATCCCAAAGTAAT
**Hat1**	F:TGATGAAAGATGGCACTACTTTCTAGT<br>R:AGCCTACGGTCGCAAAGAG
**TWF1**	F: CAGGTTAGACTCTCAGAATGCCC<br>R: CTTCAGAGTCGCTCTTGTTGCC
**CDK4**	F:TGGCTGCCACTCGATATGAAC<br>R: CCTCAGGTCCTGGTCTATATG
**PIK3AP1**	F: GTCCCGGATGCCTCTTTCTC<br>R: CACAAGTCATTTCCTGCCAGT
**APC**	F: CTACGGAAGTCGGGAAGGAT<br>R: CCTGATCTGCCTTGCTTCAT
**CDKN2B**	F: AGATCCCAACGCCCTGAAC<br>R: CCCATCATCATGACCTGGATT
